# FIDELITY AND UTILIZATION OF THE ADULT DAY SERVICES (ADS) PLUS PROGRAM

**DOI:** 10.1093/geroni/igad104.0523

**Published:** 2023-12-21

**Authors:** Katherine Marx, Laura Gitlin, David Roth, Joseph Gaugler

**Affiliations:** Johns Hopkins University, Baltimore, Maryland, United States; Drexel University, Philadelphia, Pennsylvania, United States; Johns Hopkins University, Baltimore, Maryland, United States; University of Minnesota, Minneapolis, Minnesota, United States

## Abstract

The purpose of this study is to examine intervention processes including session completion and content of the ADS Plus program. Interventionists completed an investigator-developed delivery assessment form following completion of each ADS Plus session with participating caregivers. Additionally, information on care challenges addressed and strategies offered in the form of an “ADS Plus prescription were documented. A total of 102 caregivers took part in ADS Plus. There were 954 sessions conducted, an average of 9.4 sessions per caregiver (Range 0-22). The majority of sessions were completed face-to-face (56.8%), or by telephone (40.7%). The average time spent preparing for each session by staff was 15.5 minutes (range 0 -65). Top care challenges selected by caregivers to address in ADS were: (1) Taking care of self (n=34 caregivers), (2) Meaningful activities for the person living with dementia (n=17), and (3) Agitated behaviors (n=13). Most sessions included validation and support of caregivers (91.8%) and information on caregiver self-care (85.5%). Education (49.0%) and skill enhancement for challenge areas (50.1%) were provided in about half of the sessions. Only about a quarter of sessions, (23.9%) included provision of referrals and linkages. At follow-up caregivers reported that the care challenges they sought to address in ADS Plus had gotten better (54.4%) or was eliminated (26.3%). Caregivers of persons living with dementia who attend an ADS can be aided by the addition of the ADS Plus program to address targeted caregiving needs.

